# Calibration of 0D/1D models based on OBD data for quick remapping of SI engine ECUs

**DOI:** 10.1016/j.mex.2025.103377

**Published:** 2025-05-19

**Authors:** Adrian Irimescu, Simona Silvia Merola

**Affiliations:** Science and Technology Institute for Sustainable Energy and Mobility STEMS—CNR, Via G. Marconi 4, 80125 Napoli, Italy

**Keywords:** Spark ignition engine, 0D/1D simulation, Model calibration method, ECU remapping, 0D/1D model calibration based on OBD data

## Abstract

Use of alternative energy sources in internal combustion engines requires modifying the electronic control unit (ECU) software compared to standard fuels such as gasoline or diesel. This is often called “remapping” and is usually approached through a combination of numerical and experimental techniques. In the case of spark ignition (SI) power units the new set of control settings is focused on “maps” that contain parameters such as injection duration and ignition timing; within this context, the proposed method concentrates on the use of 0D/1D modeling. A new model calibration procedure was developed for achieving acceptable accuracy by using original equipment manufacturer (OEM) ECU data acquired through the on-board diagnostic (OBD) port. The core assumption is that existing control parameters can be used for calibrating a 0D/1D model of the power unit; validation was achieved by using data recorded on two SI power units. The main advantage compared to the existing calibration procedures is that time- and cost-effective ECU remapping can be achieved with acceptable accuracy, even for fuels with properties that are quite different compared to gasoline, such as H_2_.

• 0D/1D model calibration based on OBD data recorded on an OEM ECU
• quick and cost-effective ECU remapping for using alternative fuels

## Graphical abstract

**Figure:**
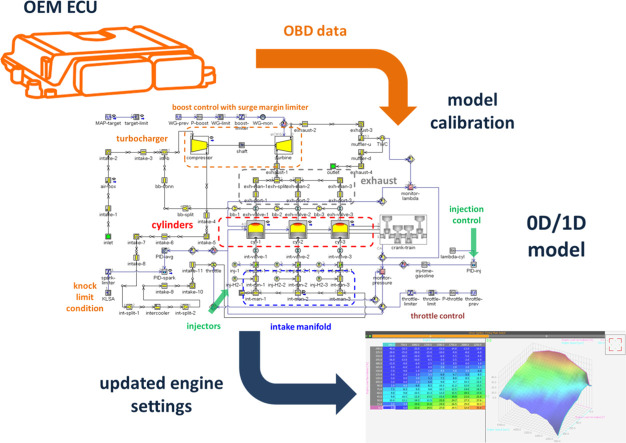

Specifications table

**Table:** 

Subject area:	Engineering
More specific subject area:	Energy and propulsion
Name of your method:	0D/1D model calibration based on OBD data
Name and reference of original method:	N/A
Resource availability:	N/A

## Background

An important part of engineering and research efforts in the field of energy and transportation is put into the calibration of models with varying levels of complexity [1,2]. The energy sector can benefit from various forms of improving existing equipment e.g. in terms of maintenance planning [3] or the design of new power units and defining required fuel properties [4]. In the specific case of combustion engines, adapting them to the use of alternative fuels can entail specific elements that are directly related to the properties of the source of energy [5,6], as well as their propensity to achieve correct operation [7]; hydrogen in particular can ensure zero-carbon emissions within a relative short timeframe [8] and in a cost-effective manner [9]. There is a definite trend of employing numerical simulation on an ever wider scale, in most steps of the design process [10]. Certain tasks can even reach the point at which most of the development is implemented in a virtual environment [11]; in such a context 0D/1D models ensure acceptable accuracy with contained computational effort. They can be implemented with a combined fundamental approach and ANN based techniques [12], while ensuring control oriented pertinence of results.

Usually the general approach is to build a model of the power unit for which the control module is to be designed or modified; in a second step, the various sub-models are calibrated based on test-bench measurements [13]. The various parts of the overall model usually correspond to physical sub-systems [14], with in-cylinder processes and combustion in particular as the most complex phenomena [15]. It is not uncommon to perform an initial analysis that is aimed at checking the coherence of overall parameters [16] and then move to combustion sub-model calibration. Model complexity depends on the level of intended accuracy, as well as the array of phenomena that need to be simulated, including stochastic cycle-to-cycle variability [17] or knocking [18]. Within this context, the current work proposes a new method of 0D/1D model calibration that will in turn be used for adapting existing spark ignition (SI) engines to alternative fuels. The starting point is the idea that data contained in original equipment manufacturer (OEM) electronic control units (ECUs) can be used for calibrating predictive combustion sub-models and thus significantly enhance simulation accuracy, while dramatically reducing costs associated with experimental trials.

## Method details

When starting the development of the method, the core assumption was that the OEM ignition settings were optimized with a certain goal in mind. Usually the process of defining these so called ECU “maps” starts with the engine setup on a test-bench; an empirical approach is employed, through which spark timing is set e.g. by assuming that combustion phasing is optimal when a certain indicator such as the peak pressure position is at a certain value [19]. This constitutes a reference or “base” setting that is stored in the ECU, and is continuously “trimmed” based on several conditions (e.g. coolant temperature and knock sensor signals). Therefore, by knowing the base ignition setting, model calibration parameters can be swept until achieving the same optimal spark advance as that acquired from the OEM ECU.

**Fig. 1:**
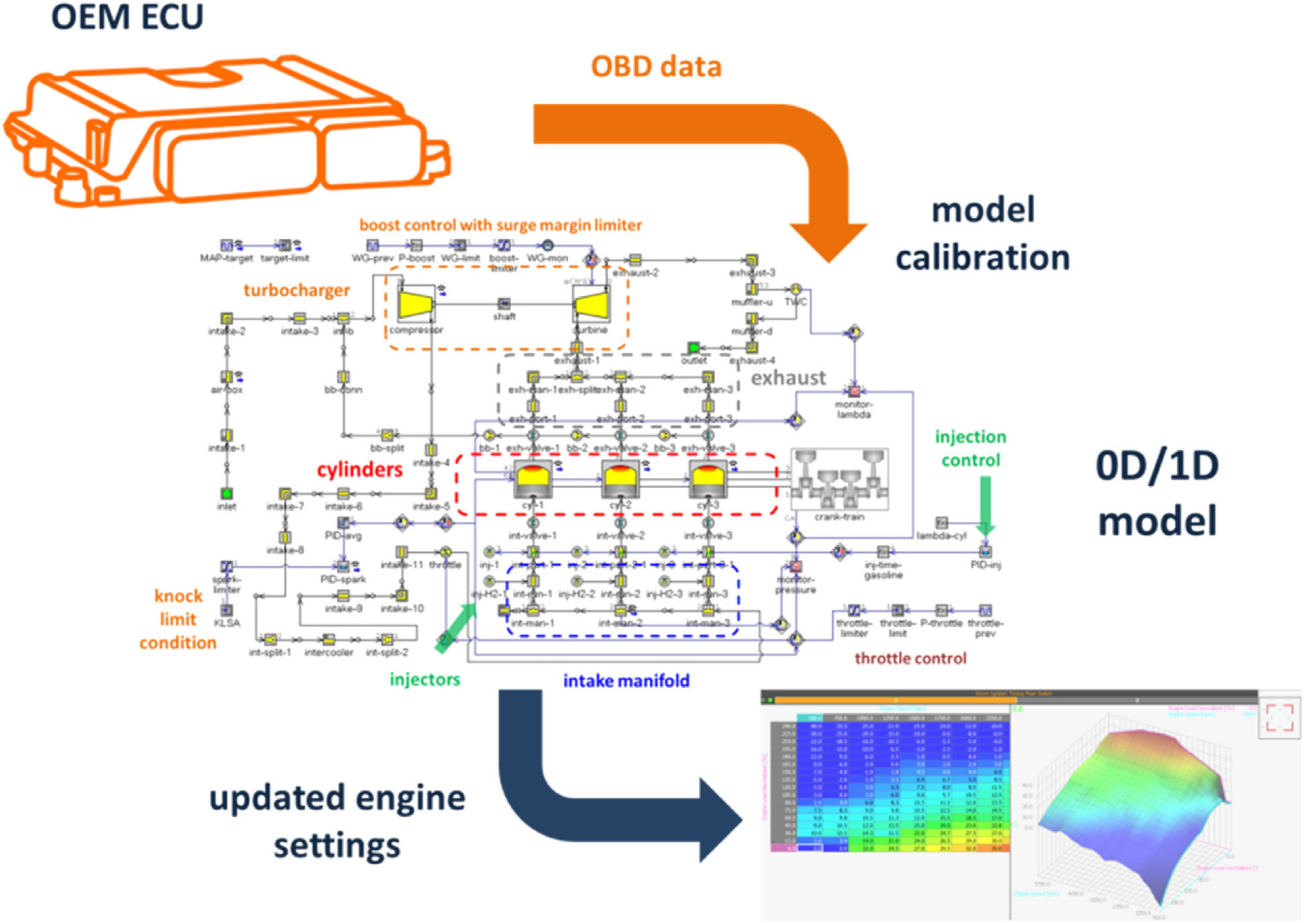
Method implementation flowchart.

Fig. 1 illustrates the general flowchart to be followed for implementing the method. Basically, it entails acquiring the ignition setting from the ECU (achievable through the on-board diagnostic (OBD) port even during normal engine operation, without the need for specific communication privileges), use these values for calibrating the 0D/1D model and then performing the simulations with the intended alternative fuel and in various operating conditions. These new maps can then be implemented in the updated ECU software version. The specifics of developing and implementing the method are further detailed in the following sub-sections, with application on two different engines.

### *Experimental setup*

**Fig. 2:**
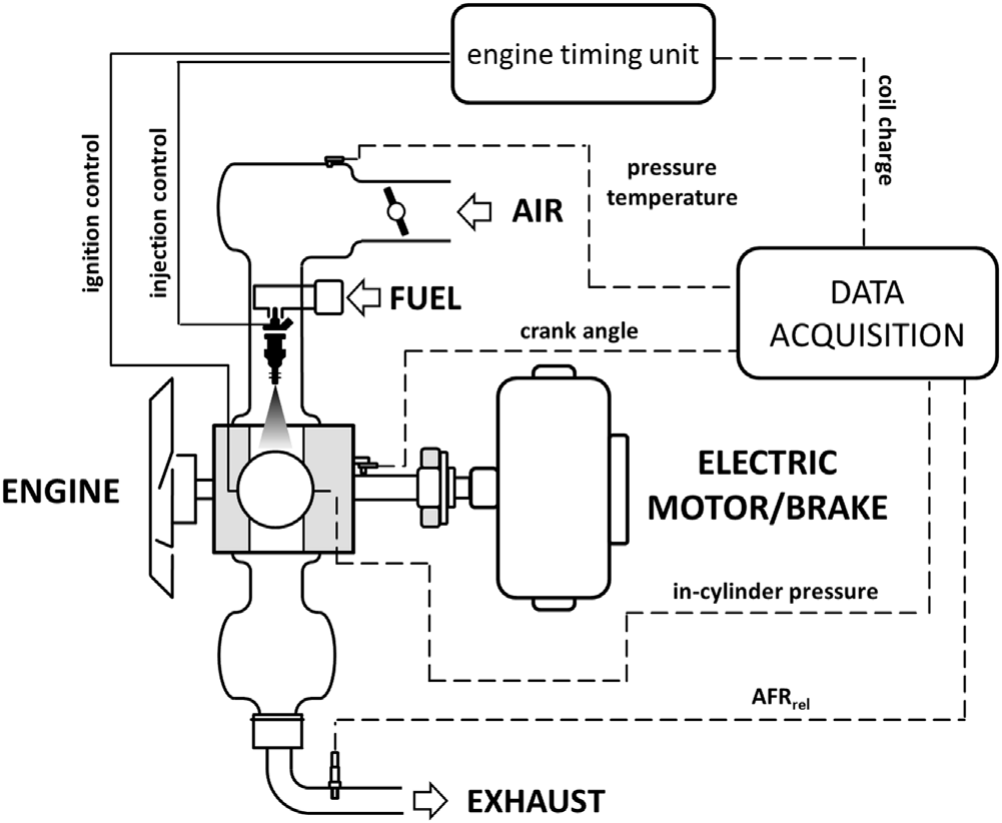
Schematic illustration of the single cylinder engine setup.

Several parameters were recorded on two SI engines, with specific focus on in-cylinder pressure traces (used for validating the simulation results). This parameter was recorded as a relative pressure value with an accuracy of ±1 % (using a piezo-electric transducer) and resolution of 0.2 deg crank angle; intake pressure was recorded as an absolute pressure value, with the same accuracy and resolution, by using a piezo-resistive sensor (this signal was used for referencing the in-cylinder traces). The single cylinder research unit (Fig. 2) employed a signal generator (with the crank angle encoder signal used as an external clock) for adjusting injection and ignition; this entailed only basic control features, but allowed complete freedom in the adjustment of spark timing settings. Several details on the setup and related procedures can be found in [20], while examples of 0D/1D modeling for this research unit can be found in [21] and [22].

The data recorded on the single cylinder engine was required for validating the core assumption; the fact that spark timing could not be controlled on the commercial power unit was another reason for this choice.

**Fig. 3:**
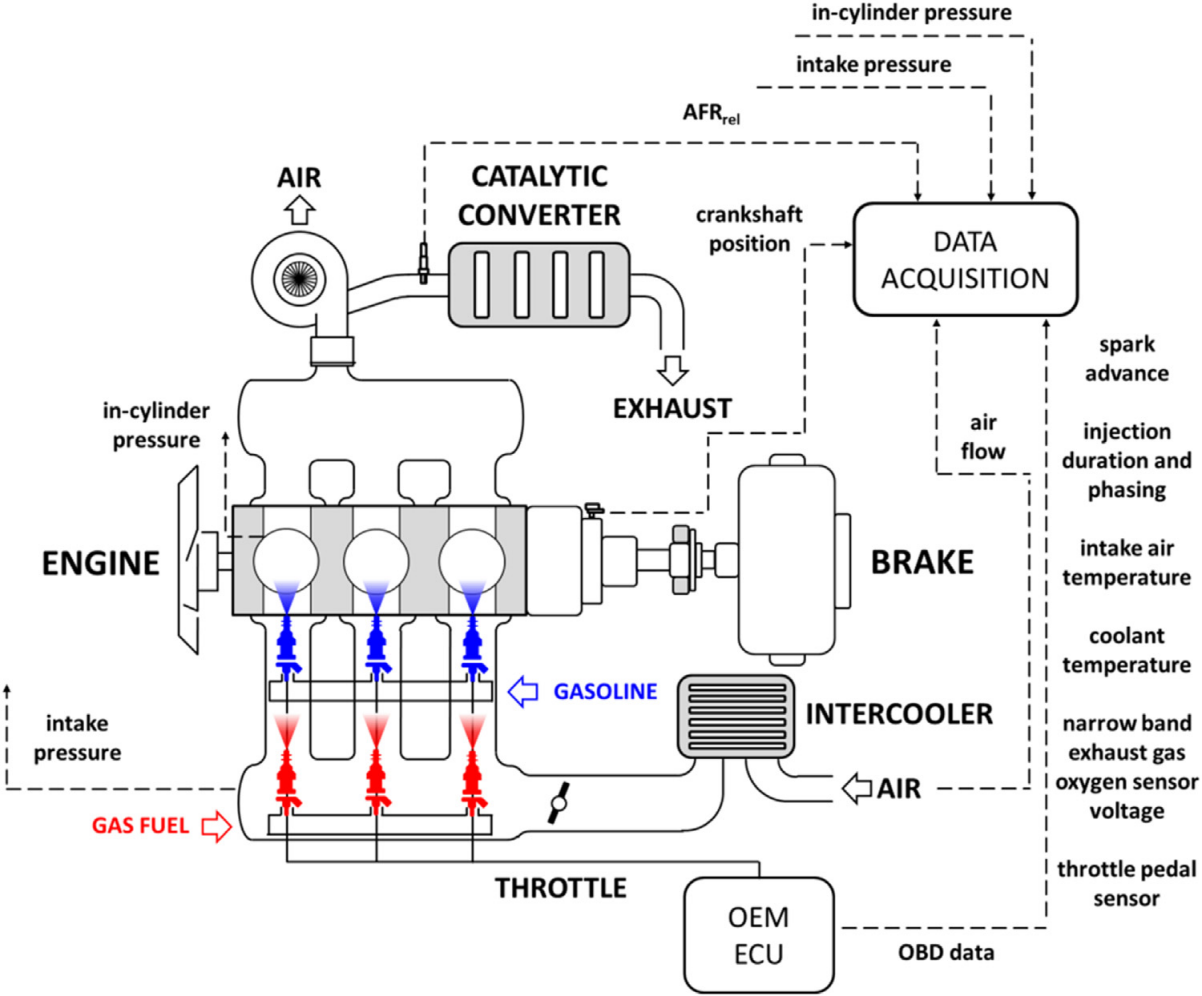
Schematic illustration of the three cylinder engine setup.

**Fig. 4:**
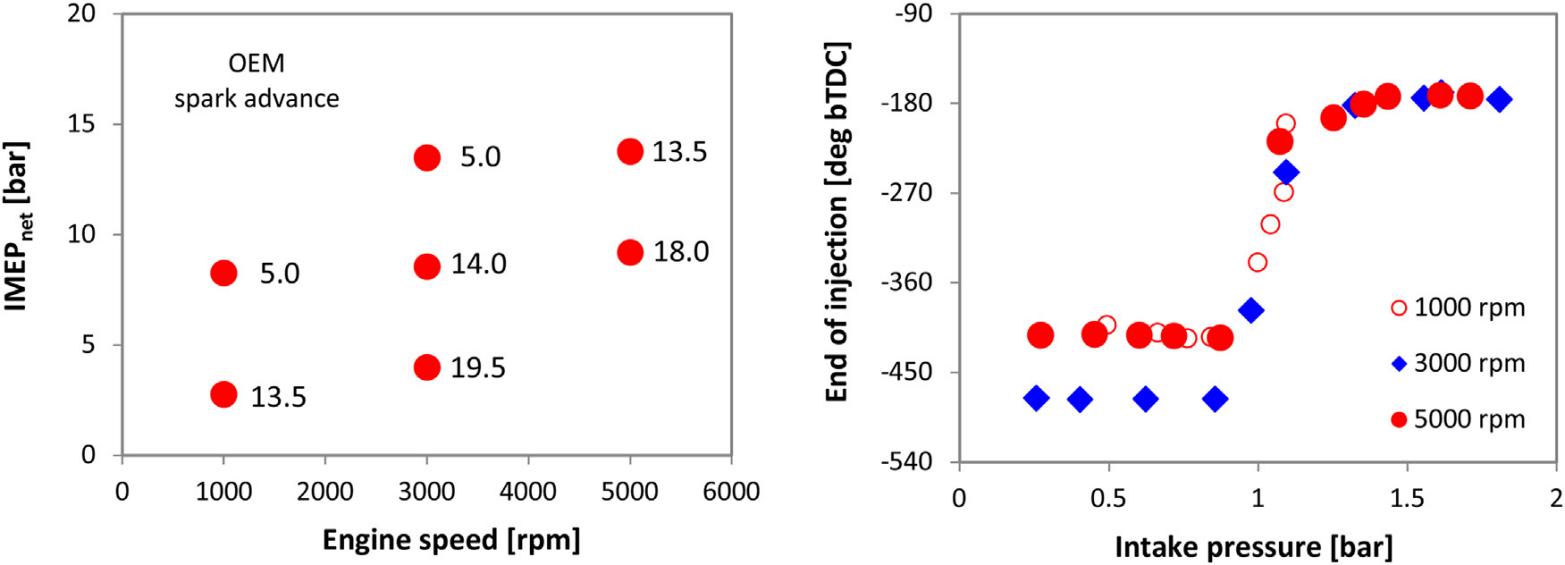
Spark advance recorded through the OBD interface in the 7 operating conditions; end of injection settings are also shown for the three rpm values.

The throttle pedal signal was the only modification that was implemented in terms of control settings for the three cylinder unit (Fig. 3). Most of the procedures used for performing the trials on the single cylinder unit were also followed for this engine as well. Of course, the main difference was that OBD data was available in this case; a total of 8 parameters (throttle pedal, engine speed, intake pressure, coolant temperature, air flow, injection duration, oxygen sensor voltage, spark timing) were monitored through a generic OBD2 interface with a sampling rate of 1 sample/s. High speed data acquisition was used for recording other parameters: in addition to in-cylinder and intake pressure (both measured with ±1 % accuracy and 0.5 deg resolution), other values such as intake air flow, injector and ignition control signals, as well as exhaust gas relative air-fuel ratio were recorded during 200 consecutive cycles at fixed engine speed-load. A total of 7 operating conditions were recorded at 1000, 3000 and 5000 rpm, with load set at low, medium and high. Fig. 4 shows the OEM spark advance for the 7 operating points, as well as the end of injection settings; these were used for defining the boundary conditions when performing the 0D/1D simulations.

### 0D/1D simulation

Models were built for the two engines by using a 0D/1D approach that basically entails defining an inlet-outlet circuit that includes several components; these are broken down into discrete volumes for which flow is defined in one direction; the cylinders are an exception, for which a 0D definition is implemented [23]. The predictive version of the combustion sub-model was employed so as to include fuel chemistry effects. Eqs. (1) to (5) illustrate the core concept of the combustion sub-model (derived from [24]) that uses turbulent flame propagation speed (*S_T_*) to determine the mass entrained by the flame front,

(1) $$S_{T}=C_{TFS}\cdot u^{\prime }\cdot \left(1-1/\left(1+C_{FKG}\cdot {\left(R_{f}/L_{i}\right)}^{2}\right)\right),$$

where *S_T_* turbulent flame speed in m/s, *u’* turbulence intensity in m/s, *R_f_* flame radius and *L_i_* integral length scale, both measured in m. The actual heat release calculation is governed by equations (2) and (3),

(2) $$dM_{b}/dt=\left(M_{e}-M_{b}\right)/\tau ,$$

(3) $$dM_{e}/dt=\rho_{u}\cdot A_{e}\cdot \left(S_{T}+S_{L}\right),$$

with *M_b_* as burned mass in kg, *M_e_* entrained mass in kg, *τ* burn-up time in s, *ρ_u_* unburned gas density measured in kg/m^3^, *A_e_* flame front surface area in m^2^ and *S_L_* as the laminar flame speed in m/s. *M_b_* is directly related to burn-up time (*τ*) and the scale of the reaction zone (*λ*), defined by Eq. (4) and (5),

(4) $$\tau =\lambda /S_{L},$$

(5) $$\lambda =C_{TLS}\cdot L_{i}/\sqrt{Re_{t}},$$

with *λ* as the Taylor length scale measured in m and *Re_t_* the turbulent Reynolds number.

Eqs. (1) through (5) also highlight the three calibration parameters *C_FKG_, C_TFS_* and *C_TLS_* (multipliers related to the flame kernel growth, turbulent flame speed and Taylor length scale respectively) that can be adjusted; a fourth one is also available in the form of the dilution exponent multiplier (*DEM*) that accounts for the presence of residual gas in the fresh charge. There is no standard procedure for calibrating the four parameters, but the manual does suggest the application of a genetic algorithm based optimizer that may provide a combination of values that best respond to a certain objective (e.g. minimizing the difference between the predicted and simulated heat release traces). For the purpose of this work, it was arbitrarily chosen to act only on the *C_TLS_* parameter. This choice was determined by the fact that a preliminary analysis of the effect of each multiplier revealed that *C_TLS_* is the one most closely connected with fuel oxidation (the process also linked with the highest level of uncertainty). The effect of the term containing *C_FKG_* was found to be fully compatible with the assumption that the flame front evolves from a laminar state to turbulent propagation during the early stages after ignition [25], and therefore the default value was kept. *C_TFS_* influences predicted heat release indirectly through the entrained mass value, and given that it was found that changes in this parameters could be relatively easily compensated through modifications of *C_TLS_* [21],it was decided to keep it at its default value as well. The fourth parameter (*DEM*) acts directly on the value of laminar flame speed and can therefore be included as an overall effect in the changes made to *C_TLS_*. Apart from the fact that the approach of acting on one parameter only can mitigate minor shortcomings that would require modifying the other multipliers, the procedure also simplifies implementation, meaning that a simple sweep of *C_TLS_* can relatively easily identify the optimal calibration value.

**Fig. 5:**
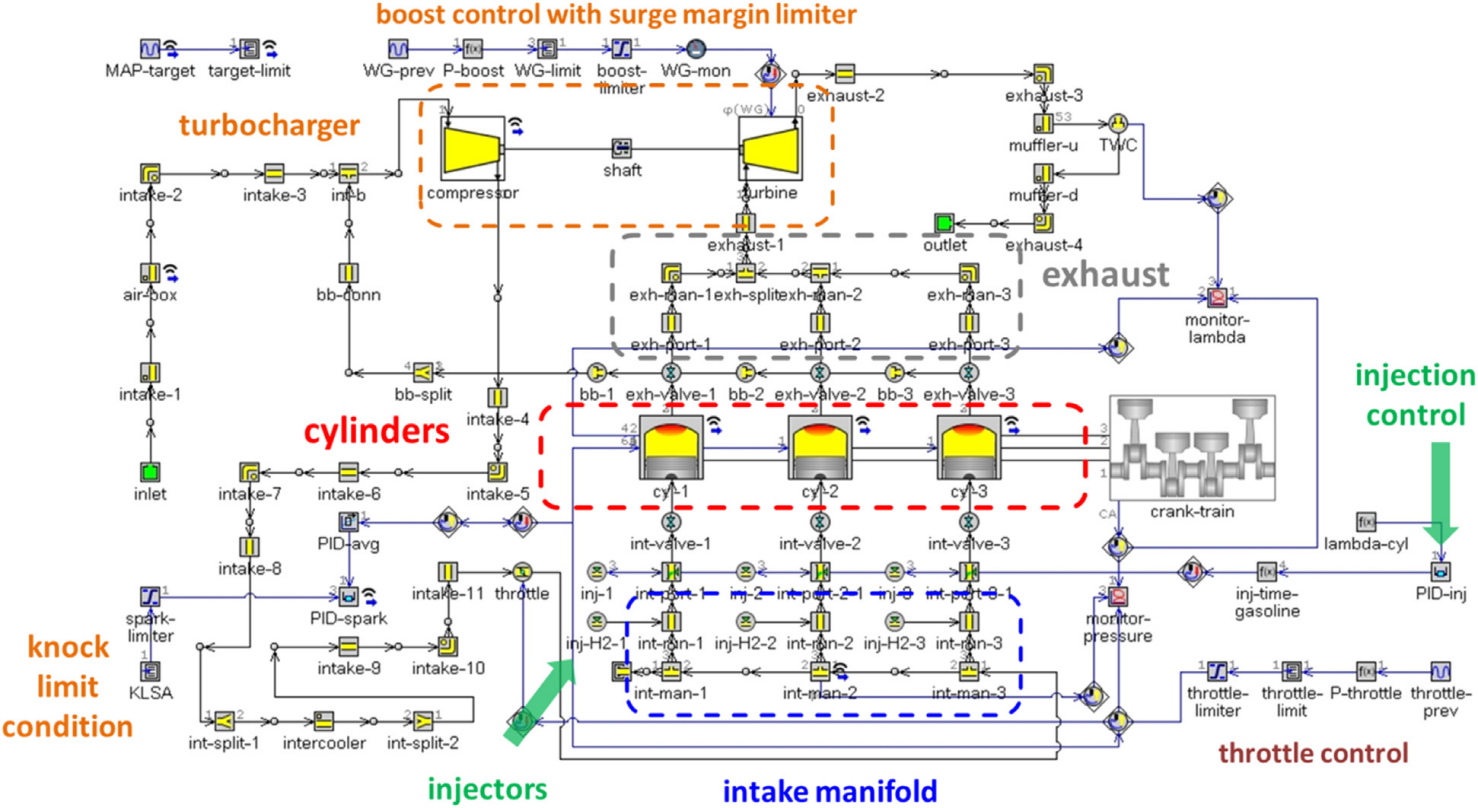
Overview of the 0D/1D model components for the three cylinder engine.

Fig. 5 shows the overall layout of the three cylinder engine model, with several parts highlighted; the control oriented approach is also illustrated, in terms of various sub-systems. For brevity, only the three-cylinder engine model is shown; the overall concept is the same for the single cylinder unit model, but with less complexity in terms of control settings. Simulations were extended to methane and hydrogen for showing an example of predicted spark timing and related ECU remapping requirements when considering conversion from gasoline fueling. The flow based heat transfer model was employed so as to correctly simulate heat transfer loss; the default settings were chosen for the flow sub-model, given that it was found to give good results compared to spark anemometry data [20]. There was no attempt to include other details such as flame instability effects [26,27]; this was mostly motivated by the fact that only stoichiometric operation was considered for the example shown here. The only modification to the combustion sub-model was to implement a dedicated laminar flame speed correlation for H_2_ representative for engine-like conditions [28]; the correlations built into the code were used for the other two fuels. The knocking sub-model of Douaud and Eyzat was used in its single-zone version just to give an idea into spark advance limitations; RON95 was employed for gasoline, while for methane and hydrogen this parameter was set at 130. The unburned mass fraction at knock onset was chosen as the feedback parameter for applying spark timing retardation through an if/then condition that acts on the PID-spark controller lower limit, when abnormal combustion is predicted.

## Method validation

Once the concept was defined and the details for implementing the method were decided, the data recorded on the two engines were used for validation. Evidently, the analysis was focused on in-cylinder pressure traces, as direct indication of correct prediction of heat release rates.

### Single cylinder engine

The single cylinder unit featured several peculiarities and related limitations not uncommon for research engines. Nonetheless, it also featured the possibility of performing a spark timing sweep that was central in validating the concept. Basically, the maximum brake torque (MBT) ignition setting was identified through measurements, and afterwards, simulations were performed for several TLS multiplier settings. In this way, a certain *C_TLS_* value could be identified as appropriate and then the predicted in-cylinder pressure trace could be compared to the measurements.

**Fig. 6:**
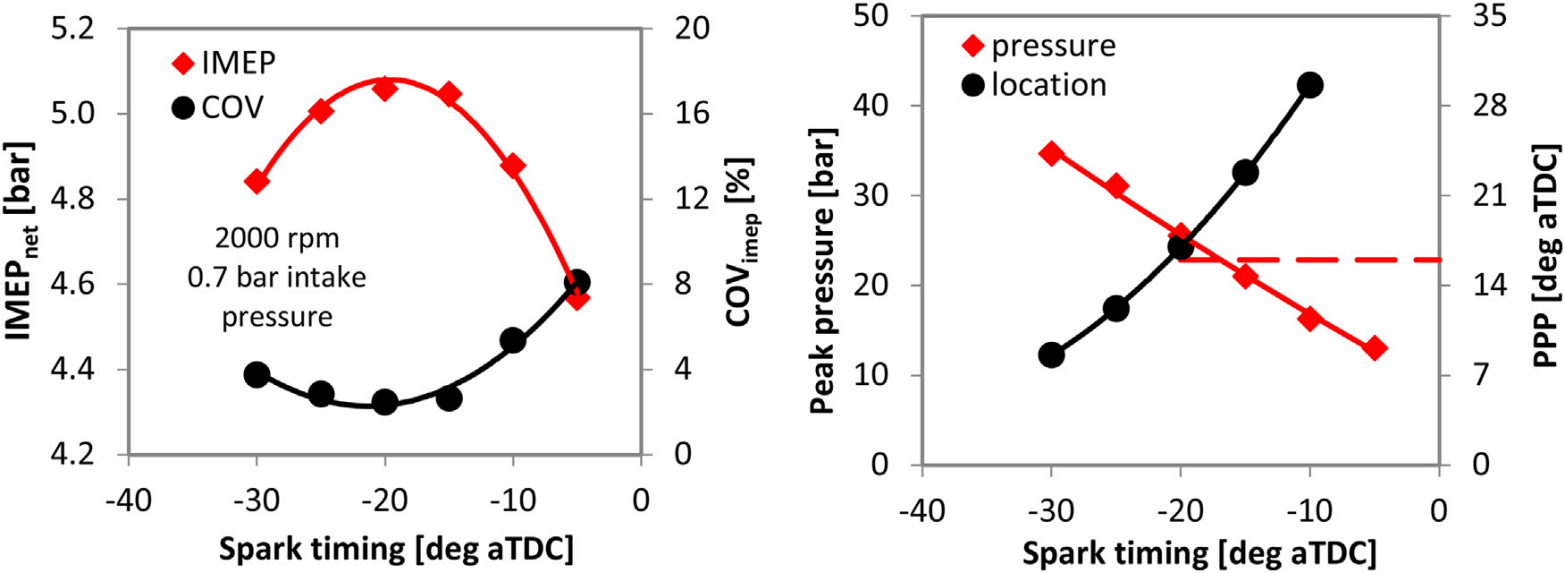
IMEP, its COV, peak pressure and its position measured on the single cylinder engine at 2000 rpm, 0.7 bar intake pressure with gasoline fueling.

**Fig. 7:**
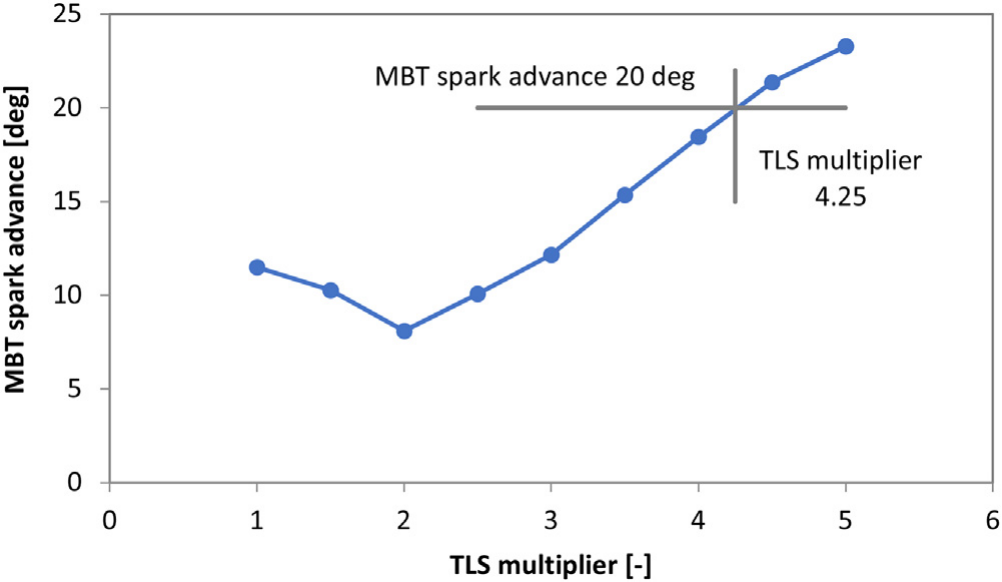
Predicted MBT for different TLS multiplier settings.

Fig. 6 shows measured IMEP, its COV, as well as peak pressure and its location with throttled operation of the single cylinder engine. Although the actual MBT setting would be in-between 15 and 20 deg spark advance, the 20 deg bTDC settings was chosen, given that it ensures a peak pressure position (PPP) close to the 16 deg marker usually considered as optimal [19] (i.e. the 17 deg PPP value for 20 deg spark advance was considered as more representative, given that the location of peak pressure was close to 23 deg aTDC for the 15 deg spark advance setting). With most of the calibration parameters kept constant (e.g. flame kernel growth and turbulent flame speed multipliers, as well as blow-by and crevice sub-model settings), the TLS multiplier was swept from its default value of 1 to 5. Fig. 7 show the results, with the measured MBT spark advance indicated along with the corresponding TLS multiplier.

**Fig. 8:**
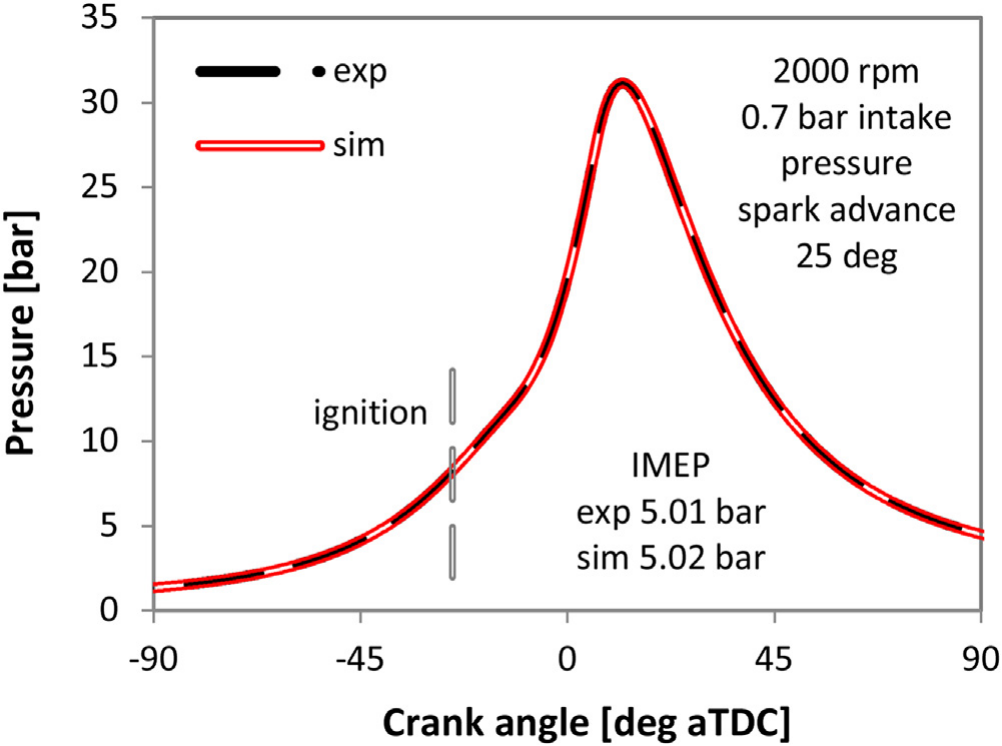
Measured (dashed line) and simulated (continuous line) in-cylinder pressure traces for 25 deg spark advance, with the 4.25 TLS multiplier.

As expected, lower TLS values predicted retarded spark timing settings, given that combustion duration was shorter; the contradicting trend in the TLS range from 1 to 2 is the result of complex interactions specific for the research engine. More to the point, “faster” combustion resulted in higher peak pressure and increased blow-by losses. Predicted flame propagation is another reason for the observed variation of predicted MBT ignition setting, given that with “slower” combustion the point at which flame propagation was terminated occurred later during the cycle and resulted in higher combustion efficiency. Basically the code switched from flame propagation mode to kinetic combustion very early when TLS was low, resulting in prediction of high unburned charge fraction at the end of the cycle. Anyway, the main conclusion is that the measured MBT spark advance of 20 deg is coupled to a value of TLS 4.25; this setting was used for simulating the case shown in Fig. 8. Very good correlation between measured and simulated traces can be noted. Given this result, it was hypothesized that by knowing the MBT ignition setting, there would be a corresponding TLS value that ensures good accuracy of the predicted in-cylinder pressure trace. This is the core idea of the procedure that was validated on the commercial engine as well.

### Three cylinder engine

Once the hypothesis that there is a correlation between optimal spark timing and combustion calibration parameters was verified on the single cylinder unit, the approach was tested on the three cylinder engine. Again, the idea was to change only the TLS parameter, in light of complex correlation between turbulence characteristics and flame interactions, as well as to keep the approach simple.

**Fig. 9:**
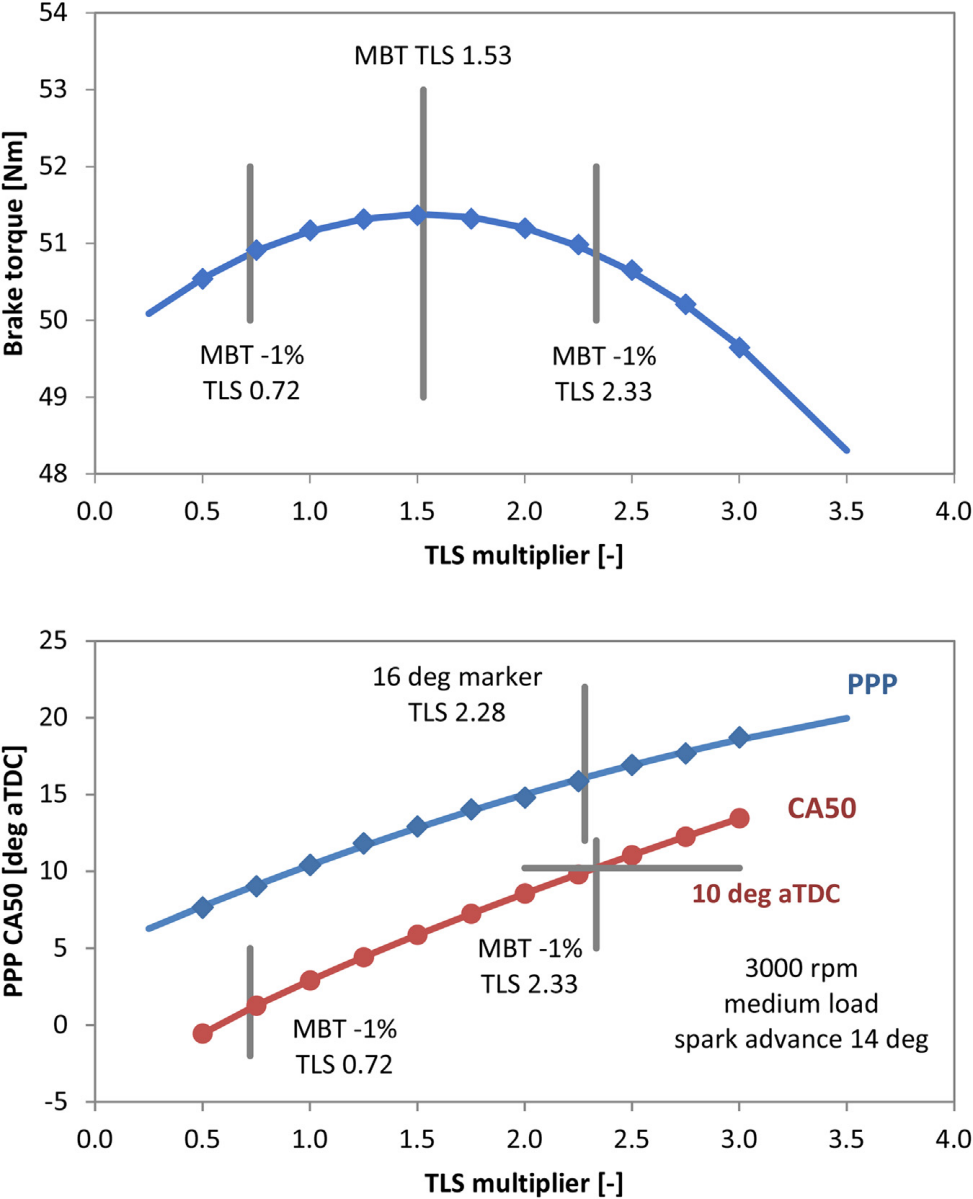
Predicted brake torque (top) and combustion phasing parameters (bottom) for 3000 rpm, medium load.

As a first step, the simulations were aimed at identifying the optimization parameter most likely to have determined the choice of the OEM spark timing settings. To this end, the engine speed and load points detailed in the previous section were taken as boundary conditions, with the ignition setting recorded through the OBD port. Fig. 9 shows the predicted brake torque, peak pressure position and the crank angle at 50 % MFB (CA50) at 3000 rpm and mid-load. The TLS sweep suggests that the optimum setting is coupled with a multiplier value around 1.5; standard practice is to retard ignition so as to obtain torque output around 1 % less than the maximum [19]. The two points that correspond to the aforementioned reduction of 1 % already seem to suggest that a value of TLS multiplier around 2.3 would be an appropriate choice for calibrating the combustion sub-model, given that it also fulfills the two most widely used empirical indicators of optimal combustion in terms of PPP at around 16 deg aTDC and 50 % mass fraction burned angle (CA50) at 10 deg aTDC [19].

**Fig. 10:**
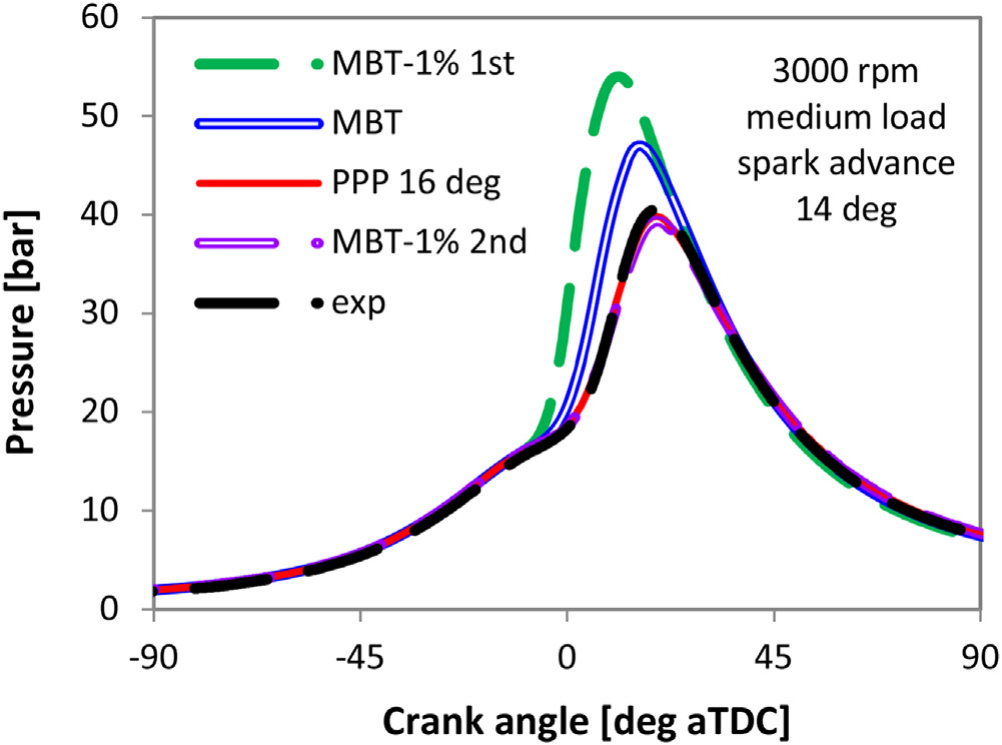
Measured (dashed dark line) and simulated in-cylinder pressure traces with different TLS settings at 3000 rpm, medium load.

Indeed, by performing the simulations with the three TLS points highlighted in Fig. 9 (i.e. MBT and −1 % MBT left-right), only the settings that are linked with the PPP 16 deg aTDC and CA50 10 deg aTDC indicators ensure an acceptable accuracy in terms of simulated in-cylinder pressure trace (Fig. 10).

**Fig. 11:**
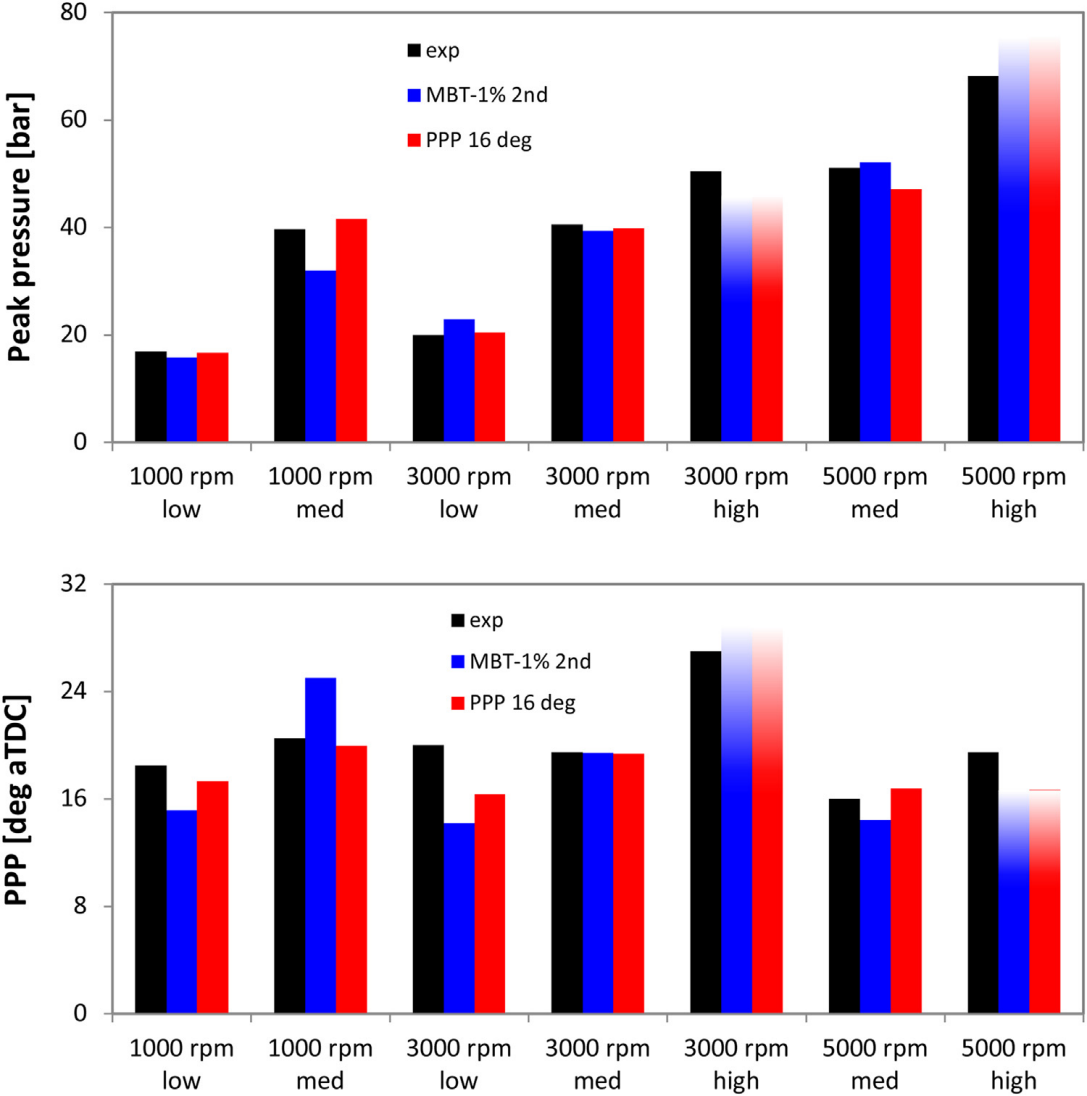
Comparison of simulated and measured peak pressure (top) and its position (bottom) with two different TLS multiplier settings; transparency effect was added for the two conditions with possible knocking.

To extend the verification further, numerical results were compared to the measurements in terms of maximum in-cylinder pressure and its location. Fig. 11 shows the results obtained for all 7 engine speed-load points that were chosen. The comparison suggests that acceptable results can be obtained by applying the proposed calibration method. No clear advantage could be identified in terms of choosing the CA50 or PPP as the optimization target; setting the PPP at 16 deg aTDC as a condition seems to provide results closer to the measurements, but overall the two approaches are comparable.

### Verification of predicted spark advance and extension to alternative fuels

Limited datasets were available with in-cylinder pressure measurements and so these were used only for verifying the core hypothesis. OBD data only instead, featured more statistically relevant spread and allowed the evaluation of actual feasibility of applying the new method for remapping ECUs.

**Fig. 12:**
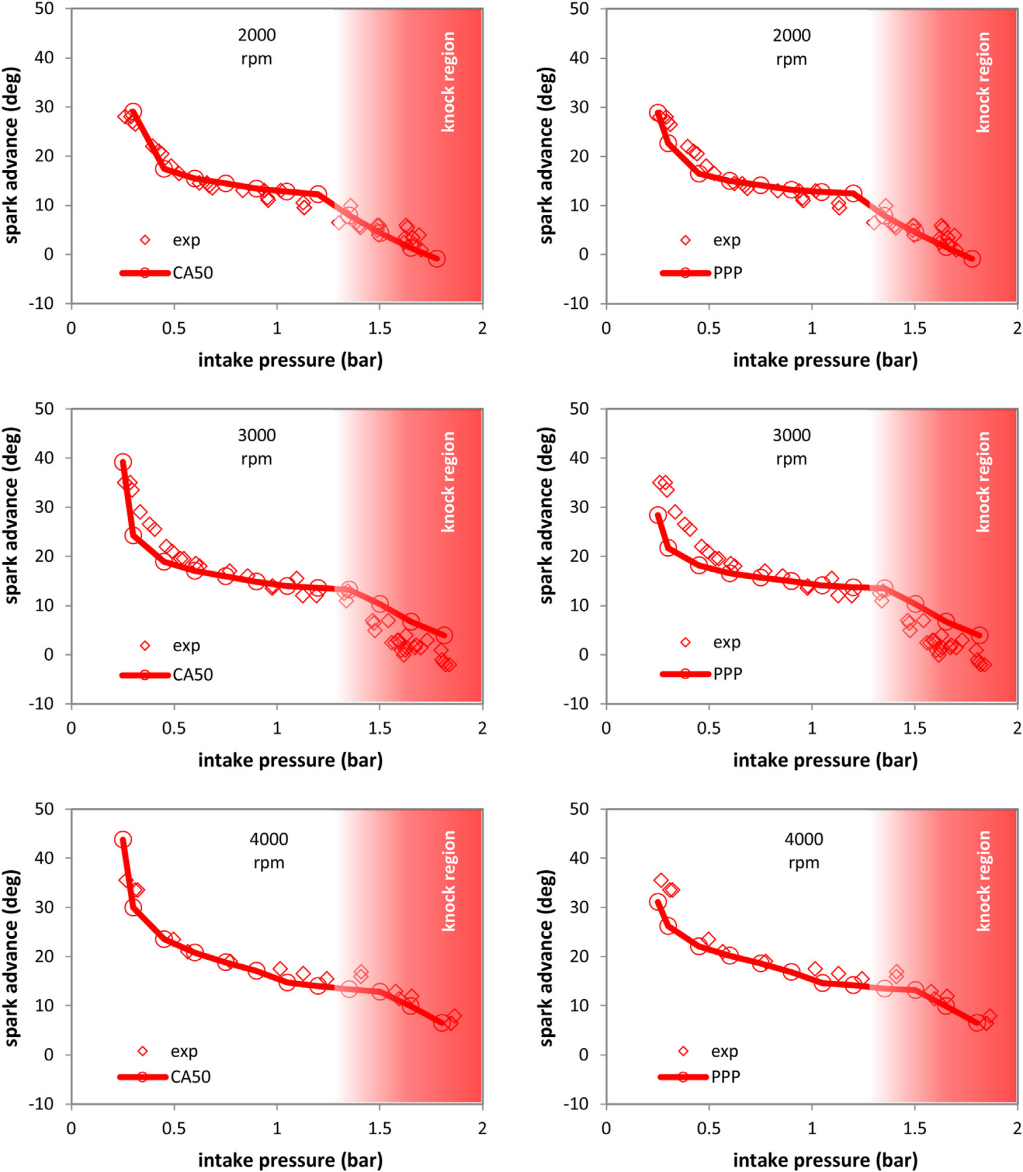
Recorded (symbols) and predicted (symbols with lines) spark advance at 2000, 3000 and 4000 rpm (from top to bottom), when using the CA50 at 10 deg aTDC (left) and the PPP at 16 deg aTDC assumption (right).

Fig. 12 shows measured and predicted spark timing settings throughout the load range at three different rpm values. The model correctly predicted the steep optimal spark advance variation in the low load region and the relatively flat trend in mid-load conditions. Knock prone regions are highlighted with a red transparent rectangle. Although no knocking was recorded during the measurements (e.g. at 3000 rpm, high load), the code did predict that such phenomena are probable. A threshold of 15 % MFB at knock onset was chosen as the value to be used by the knock limited spark advance (KLSA) module; this sub-model set a certain limit for spark timing if knocking was predicted. The threshold value was chosen based on the fact that with the OEM ignition setting and calibrated TLS the model would predict 15 % unburned charge at knock onset for the condition of 1000 rpm and mid-load.

Even if the threshold is somewhat high, it was deemed as an acceptable choice for implementing this part of the model. Indeed, the model seems to slightly underestimate this effect at 3000 rpm, but predicted correctly the ignition settings trend for 2000 and 4000 rpm. Both optimization parameters (i.e. PPP and CA50) delivered comparable results. The CA50 choice seems to perform slightly better and for this reason, it was chosen for comparing predicted methane and hydrogen mapping requirements at 3000 rpm.

**Fig. 13:**
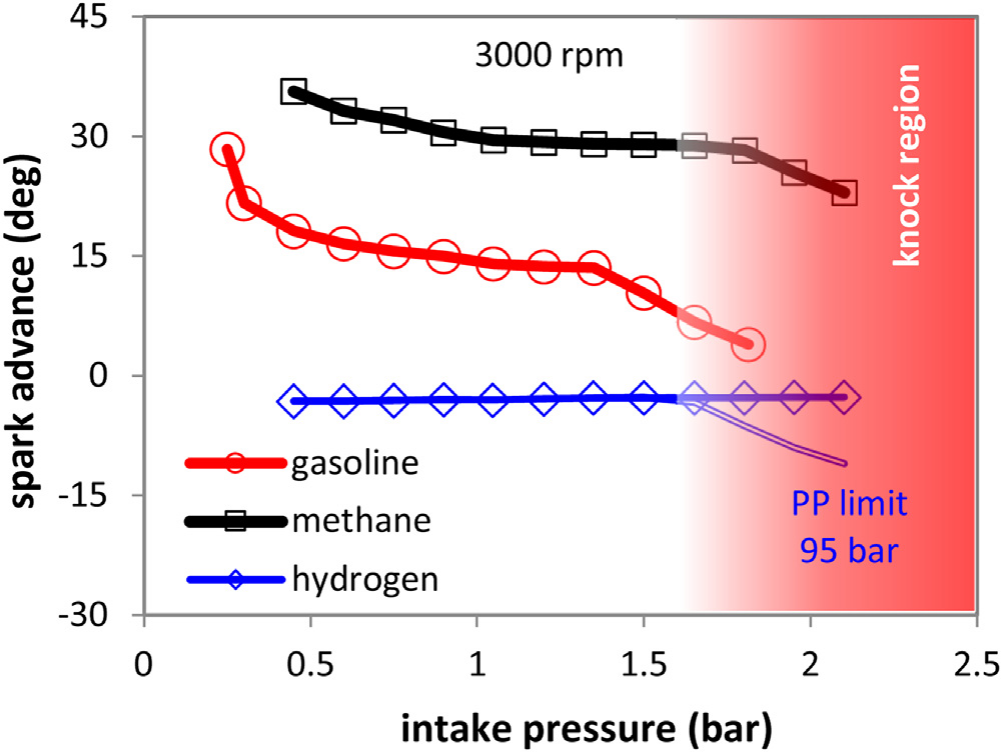
Predicted spark advance at 3000 rpm with gasoline (round symbols), methane (square symbols) and hydrogen fueling (diamond symbols); predictions with a 95 bar peak pressure limit are also shown with a double line.

Fig. 13 shows the results obtained throughout the load range for the three fuel types. It emphasizes the need to increase boosting (already identified as essential when substituting gasoline with gaseous fuels [29]) and thus the final rpm-intake pressure map would have to be extended for methane and hydrogen compared to gasoline. As expected, given the lower laminar flame speed of methane, spark timing needs to be advanced compared to gasoline. On the contrary, for H_2_ much more retarded settings would need to be implemented, given its very high laminar flame speed.

Peak pressure (PP) limitations were also noted to be a possible issue for engines converted to H_2_ [30,31]; simulations were performed with an 95 bar PP limit (chosen based on the predicted peak pressure with gasoline fueling and observed variability during high load operation) and the model predicted a trend of retarded ignition settings, similar to the other two fuel types when increasing boost pressure. It should be noted that the underlying reason is different, meaning that gasoline and methane were knock limited, while H_2_ was peak pressure limited.

## Limitations

One of the main limitations of the method is that it is based on the hypothesis that the spark timing is directly linked with the MBT setting. This may not the case for certain situations such as cold start, catalyst heating mode or other specific situations that entail spark advance trimming. This shortcoming can be mitigated with a certain level of know-how that the user can employ for choosing the operating conditions that are most likely to comply with the aforementioned core hypothesis, and then proceed with model calibration.

The fact that the method relies on the predictive entrainment combustion sub-model is another limitation, meaning that results may not be accurate when considering fuels such as H_2_ and ultra-lean conditions. One positive aspect is that the model is only part of the method; accuracy could be improved by choosing a different combustion sub-model (i.e. calibration is most likely approachable in the same way with other simulation tools) that correctly captures phenomena such as intrinsic flame instabilities.

Another aspect is that the method is directly linked with specifics of automotive type ECUs; this however should be of minor influence, given that most off-road OEMs follow roughly the same development process for implementing control strategies.

## CRediT author statement

**Adrian Irimescu:** Conceptualization, Methodology, Validation, Writing – Original draft. **Simona Silvia Merola:** Writing – Review & Editing, Visualization.

## Declaration of competing interest

The authors declare that they have no known competing financial interests or personal relationships that could have appeared to influence the work reported in this paper.

## Data Availability

Data will be made available on request.
